# A Potential Prognosis Indicator Based on P300 Brain–Computer Interface for Patients with Disorder of Consciousness

**DOI:** 10.3390/brainsci12111556

**Published:** 2022-11-15

**Authors:** Jingcong Li, Biao Huang, Fei Wang, Qiuyou Xie, Chengwei Xu, Haiyun Huang, Jiahui Pan

**Affiliations:** 1 School of Software, South China Normal University, Guangzhou 510631, China; 2 Pazhou Lab, Guangzhou 510631, China; 3 Joint Research Centre for Disorders of Consciousness, Department of Rehabilitation Medicine, Zhujiang Hospital of Southern Medical University, Guangzhou 510631, China

**Keywords:** P300, BCI, DOC, prognosis, CRS-R

## Abstract

For patients with disorders of consciousness, such as unresponsive wakefulness syndrome (UWS) patients and minimally conscious state (MCS) patients, their long treatment cycle and high cost commonly put a heavy burden on the patient’s family and society. Therefore, it is vital to accurately diagnose and predict consciousness recovery for such patients. In this paper, we explored the role of the P300 signal based on an audiovisual BCI in the classification and prognosis prediction of patients with disorders of consciousness. This experiment included 18 patients: 10 UWS patients and 8 MCS- patients. At the three-month follow-up, we defined patients with an improved prognosis (from UWS to MCS-, from UWS to MCS+, or from MCS- to MCS+) as “improved patients” and those who stayed in UWS/MCS as “not improved patients”. First, we compared and analyzed different types of patients, and the results showed that the P300 detection accuracy rate of “improved” patients was significantly higher than that of “not improved” patients. Furthermore, the P300 detection accuracy of traumatic brain injury (TBI) patients was significantly higher than that of non-traumatic brain injury (NTBI, including acquired brain injury and cerebrovascular disease) patients. We also found that there was a positive linear correlation between P300 detection accuracy and CRS-R score, and patients with higher P300 detection accuracy were likely to achieve higher CRS-R scores. In addition, we found that the patients with higher P300 detection accuracies tend to have better prognosis in this audiovisual BCI. These findings indicate that the detection accuracy of P300 is significantly correlated with the level of consciousness, etiology, and prognosis of patients. P300 can be used to represent the preservation level of consciousness in clinical neurophysiology and predict the possibility of recovery in patients with disorders of consciousness.

## 1. Introduction

Disorders of consciousness (DOC) are severe sequelae of brain injury characterized by deficits in consciousness and cognitive impairment, including coma, unresponsive wakefulness syndrome (UWS, also known as a vegetative state), minimally conscious state (MCS), and locked-in syndrome (LIS). Patients with UWS do not have discrete localized motor control, cannot express comprehensible words, and cannot open their eyes spontaneously to obey verbal commands [1]. It is possible for them to wake up but not be aware of themselves or the environment. In contrast, patients with MCS are characterized by inconsistent but reproducible signs of consciousness through responses [2,3]. In addition, a feature that appears in MCS (EMCS) is reliable and consistent functional interaction communication or demonstration of functional usage of two different objects [3]. Recently, this term has been divided into minimally conscious state minus (MCS-) and minimally conscious state plus (MCS+) based on the level of nonreflexive responsiveness of these patients, and this subcategorization has been validated with [18F]-fluorodeoxyglucose positron emission tomography (FDG-PET) showing distinct patterns of cerebral metabolism across the two categories (MCS- and MCS+) [4,5,6,7]. MCS- is characterized by a response to meaningful stimuli (e.g., visual pursuit, localization of noxious stimuli, and/or smiling/crying in relation to external stimuli), while MCS+ is characterized by command following. LIS patients are characterized by dysarthria and quadriplegia, and their cognitive abilities are largely preserved [8]. Several studies have not included LIS as a DOC [9]. Different patients have different levels of consciousness. Accurate assessment of patients’ consciousness is key to diagnosis and treatment by physicians and caregivers. The current clinical diagnosis of consciousness in patients is based on behavior scales, such as the JFK Coma Recovery Revised-Scale (JFK CRS-R), which relies on the patient’s motor response to external stimuli to assess the patient’s level of consciousness [10]. The JFK CRS-R is considered to be a reliable and widely applied clinical assessment [11]. It was first introduced by Giacino et al. in 1991 and was revised in 2004 [12]. The JFK CRS-R contains six subscales, including auditory, visual, motor, oromotor, communication, and arousal functions. The scores for each subscale depend on whether patients with DOC have specific behavioral responses to sensory stimuli. However, this behavior-based assessment method has some problems [13] and is prone to misdiagnosis, especially in the assessment of UWS and MCS. First, many DOC patients cannot maintain a stable state during the process of consciousness detection, and they are more prone to fatigue or unable to cooperate with relevant detection autonomously. Second, these patients are usually unable to perform normal body movements and verbal expressions [14]. Therefore, it results in a high rate of clinical misdiagnosis. For example, in a study of 137 patients, 24.7% of patients with clinical UWS assessed on a single CRS-R behavioral assessment were actually MCS patients, and the repeated CRS-R assessment results showed a 38.2% misdiagnosis rate for MCS [15]. In recent years, there has been a lot of research on the application of biological signals based on disease prediction. In [16], Hussain et al. proposed HealthSOS, a real-time health monitoring system for stroke prognosis. The experimental results showed that the revised brain symmetry index, the delta-alpha ratio, and the delta–theta ratio of the stroke group were significantly different from that of the healthy control group. Furthermore, in [17], the authors proposed Big-ECG, a cyber-physical cardiac monitoring system for stroke management. Through experiments, they found biomarkers such as Electrocardiogram (ECG) benchmark features that were significantly different between the stroke group and the healthy group. Hussain et al. also quantified EEG features to understand task-induced neurological decline due to stroke and assessed biomarkers to differentiate ischemic stroke groups from healthy adult groups [18]. The above studies were designed to examine significant differences between the stroke group and healthy individuals. These studies also confirmed that there is indeed some significant difference between patients and healthy people, which laid the foundation for our research.

Considering these limitations of behavioral assessment scales, it is imperative to explore a non-behavior-based and objective method to diagnose patients’ consciousness and assess the clinical prognosis of patients with DOC. Recently, several studies have shown that brain–computer interfaces (BCIs) have great potential in detecting residual consciousness and predicting prognosis in DOC patients. Moreover, the type of brain injury, P300 Electroencephalogram (EEG) response, and accuracy can predict the prognosis of DOC patients to a certain extent [19,20,21,22]. Pan et al. reported a visual hybrid BCI that combined P300 and SSVEPs to detect consciousness in 4 patients with UWS, 3 with MCS, and 1 with LIS [23]. They successfully demonstrated command tracking in three patients. They proposed a hybrid system based on P300 and SSVEPs, and their results show that the performance of the hybrid BCI system is significantly improved over that of the unimodal BCI system. Overall, the performance of BCIs designed for patients with DOC is generally poor. This is mainly because DOC patients have a lower cognitive level and have a harder time concentrating. Moreover, to date, no specific or standardized prognostic indicators have been constructed to predict the probability of recovery of consciousness in patients with DOC and demonstrate the limitations of treatment in patients with brain injury.

In this paper, our main contributions are:

(**1**) We utilized a traditional Bayesian method and CNN model to detect and analyze P300 signals in DOC patients.
(**2**) We found a positive linear correlation between P300 detection accuracy and CRS-R score which indicated that the patients with the P300 detection accuracy could be used to assess patients’ state of consciousness.
(**3**) The experiments demonstrated that patients with higher P300 detection accuracies tend to have higher CRS-R score after three months, which indicated better prognosis.

## 2. Materials and Methods

### 2.1. Subjects

This study involved 18 patients with disorders of consciousness (10 “improved” and 8 “not improved”; mean age ± standard deviation (SD): 36.56 ± 10.83 years old; detailed information is shown in Table 1) at the General Hospital of Guangzhou Military Command of People’s Liberation Army, China, between October 2014 and August 2018. Their clinical diagnosis was based on the CRS-R, which comprises six subscales for auditory, visual, motor, oromotor, communication, and arousal functions. The inclusion criteria included: (**a**) a diagnosis of UWS or MCS, with no detectable command-following behavior observed during the week of admission; (**b**) more than 1 month since brain injury; and (**c**) no history of impaired vision or hearing.

The exclusion criteria included (**a**) current neuromuscular function blockers or sedation; (**b**) persistent acute illness or progressive systemic or neurological disease; and (**c**) fewer than two complete clinical assessments with the CRS-R throughout the study.

All patients were subjected to two CRS-R assessments: one week before the start of the experiment and another 3 months later. For all patients, we used the CRS-R score to judge the levels of consciousness. An increase in the CRS-R score represents a trend towards an improvement in the level of consciousness. This study was approved by the Ethics Committee of the General Hospital of Guangzhou Military Command in Guangzhou and complied with the Code of Ethics of the World Medical Association (Declaration of Helsinki). In addition, the legal surrogates of all of the patients provided written informed consent.

### 2.2. Behavioral Assessment

The cognitive function of patients with DOC was assessed by the Coma Recovery-Revised Scale (CRS-R). It is a scale used to characterize the level of consciousness and monitor neurobehavioral recovery. It includes 6 subscales covering auditory, visual, motor, oromotor, communication, and arousal functions. These diagnostic results were discovered after long-term observation by multiple specialized clinical staff. The type of diagnosis (UWS/VS or MCS) in patients with DOC was determined by the CRS-R score.

### 2.3. Paradigm

The paradigm used in this paper is the audiovisual paradigm. Specifically, two buttons are located on the left and right sides of the GUI, displaying two randomly selected Arabic numerals from 0 to 9. The two numbers’ buttons flashed in an alternating pattern, where the color of the flashing button changed from green to black and the color of the corresponding number changed simultaneously from black to white. Simultaneously read the corresponding numbers from the speakers located on the same side of the display. In this way, subjects were presented with temporally, spatially, and semantically consistent audiovisual stimuli, each lasting 300 ms, to evoke P300 responses.

### 2.4. Procedure

For BCI experiments, we collected EEG signals through a 30-channel cap (LT37) using a NuAmps device (Compumedics Neuroscan, Inc. TX, USA.). EEG signals from all electrodes were referenced to the right mastoid at a sampling rate of 250 Hz and digitized. Electrode impedance was kept at 5 KΩ. For the audio-visual paradigm-related P300, the EEG signal was first filtered from 0.1 to 10 Hz. For each flash of the frame around the digit on the screen, we obtained a segment of the EEG signal from each channel (0–600 ms after the frame flash) and downsampled this segment at a rate of 5. Then, we concatenate down-sampled segments of 10 channels (Fz, Cz, P7, P3, Pz, P4, P8, O1, Oz and O2) to obtain the data vector for each flash. Through the above-mentioned band filtering and feature extraction methods, the influence of 50 Hz, ECG, and EMG artifacts of AC power is removed. Furthermore, considering that P300 is mainly associated with activity in the delta band [24], these above artifacts do not have much impact on our P300 classification work. According to our results on 18 subjects, the preprocessing method we used is robust. For each stimulus, after bandpass filtering (0.1–20 Hz), EEG epochs for each channel were obtained from 50 ms pre-stimulus to 600 ms post-stimulus, and baseline-corrected from data from the 50 ms pre-stimulus interval. For each channel, we averaged the EEG epochs of all target and non-target stimuli to obtain two ERP waveforms. After preprocessing and feature extraction, our data basically reach class balance (details are shown in Table 2). We put the data (P300 and non-P300) as input into the network for the next step.

### 2.5. Convolutional Neural Network (CNN)

The P300 signal is difficult to detect due to its low signal-to-noise ratio. Previous studies on P300 detection and P300-based spellers have used traditional machine learning approaches, namely hand-designed signals processing techniques for feature extraction, and classifiers such as support vector machines (SVM) and linear discriminant analysis (LDA) [25]. Unfortunately, such methods limit the potential of hand-designed feature extraction and traditional classification techniques to further improve accuracy. In recent years, researchers have begun to use convolutional neural networks (CNNs) for P300-based BCI designs [26], which have achieved better accuracy than traditional methods. Therefore, in our experiments, we tried to use a CNN on a continuous segment of EEG signals (including P300 and non-P300) from 18 patients. The EEG signal with a P300 response was marked as 1, and the EEG signal without a P300 response was marked as 0. Through the learning of the CNN model, the detection accuracy of P300 is obtained.

**Figure 1:**
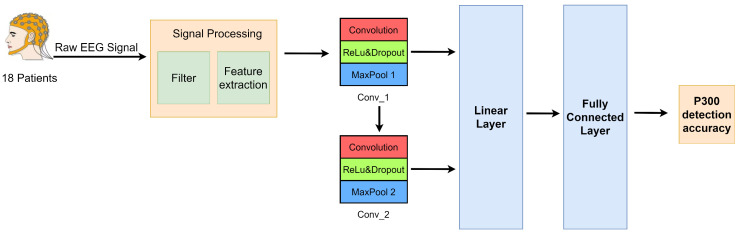
Conceptual diagram of CNN processing EEG signals.

In the experiments, we used 30 channels. In the CNN model, we employ spatiotemporal 2D convolution for feature fusion. CNN consists of five main layers, an input layer, two convolutional layers, a linear layer and a fully connected output layer. The features extracted in the previous step are provided as input to the CNN. The two convolutional layers are Conv_1 and Conv_2 based on spatiotemporal filtering. These two layers perform 2D convolutions on the channels. Linearly normalize the outputs of Conv_1 and Conv_2 layers. A Rectified Linear Unit (ReLU) is used as the activation function. Dropout is added to the network as a regularization technique to prevent overfitting. Dropout and normalization have been shown to improve the generalization performance and training speed of neural networks [27]. The output layer of the network is equipped with a softmax function to output the probability that a given input segment belongs to a particular class. The performance of the method was evaluated based on a 10-fold cross-validation scheme on each subject’s mixed P300 and non-P300 EEG data. Finally, the P300 detection accuracy is obtained. Our CNN network parameter information includes: (1) Batch_size = 16; (2) Weight_decay = 0.0001; (3) Drop_rate = 0.3; (4) Learning_rate = 0.00004; (5) epochs = 100; and (6) Optimizer: Adam. The conceptual diagram of CNN processing EEG signals is shown in Figure 1.

### 2.6. Bayesian Network (BN)

Bayesian network is also known as belief network or directed acyclic graph model. It is a structured knowledge representation in which domain variables are treated as nodes in a graph, whose structure encodes the dependencies among them. The BN learning task in Bayesian networks can be divided into two subtasks: structure learning and parameter estimation. BN structures aim to identify the optimal topology and imply a set of conditionally independent relationships between the variables involved, as long as these variables are valid [28].

In this paper, we refer to the method proposed by Blundell et al. [29]. During training, the Bayesian layer attempts to introduce uncertainty in its weights by sampling its weights from a distribution parameterized by the trainable variables on each feedforward operation. This allows us to not only optimize the performance metrics of the model, but also to collect the uncertainty of the network predictions at specific data points and to aim to reduce the variance of the network as much as possible through the predictions. Weight sampling on the Bayesian layer, we sample the parameters of the linear transformation in each feedforward operation using the following equation (where ρ parameterizes the standard deviation and μ parameterizes the mean of the sample linear transformation parameters):

For the weights:

(1) $$W_{\left(n\right)}^{\left(i\right)}=N\left(0,1\right)*\log\left(1+\rho^{\left(i\right)}\right)+\mu^{\left(i\right)}$$

where the sampled *W* corresponds to the weights used on the linear transformation for the ith layer on the nth sample.

For the biases:

(2) $$b_{\left(n\right)}^{\left(i\right)}=N\left(0,1\right)*\log\left(1+\rho^{\left(i\right)}\right)+\mu^{\left(i\right)}$$

where the sampled *b* corresponds to the biases used on the linear transformation for the *i*th layer on the *n*th sample.

### 2.7. Evaluation

Since our patients’ data are not very large, we evaluate the performances of our P300 classification using the The area under the precision–recall curve (AUPRC), Precision, Sensitivity, Specificity, F1_score F${}_{0.5}$, F${}_{2}$ and negative predictive value (NPV):

(3) $$Precision=\frac{TruePositive}{TruePositive+FalsePositive}$$

(4) $$Sensitivity=\frac{TruePositive}{TruePositive+FalseNegative}$$

(5) $$Specificity=\frac{TrueNegative}{TrueNegative+TruePositive}$$

(6) $$Recall=\frac{TruePositive}{TruePositive+FalseNegative}$$

(7) $$F1\_score=\frac{2}{\frac{1}{Precision}+\frac{1}{Recall}}$$

(8) $$F_{\beta }=\frac{(1+\beta^{2})\times TruePositive}{\left(1+\beta^{2}\right)\times TruePositive+\beta^{2}\times FalseNegative+FalsePositive}$$

(9) $$NPV=\frac{TrueNegative}{TrueNegative+FalseNegative}$$

where True Positive is the number of “positive” (i.e., P300 signal) result retrieved by the classifier; True Negative is the number of “negative” (i.e., non-P300 signal) result not retrieved by the classifier; False Positives are the classifier that incorrectly retrieved the number of “negative” results found; False Negatives are the number of “positive” results not retrieved by the classifier [30]. The specific results of 18 patients are shown in Table 3, Table 4, Table 5 and Table 6.

### 2.8. Statistics

The statistical relationship between the transition in consciousness states and the detection accuracy of P300 in the audiovisual paradigm was determined using Student’s *t*-test. According to the detection accuracy of P300 in the audiovisual paradigm, the ERP results were divided into two types. The other is the state of the patient after three months. When followed up with three months later, patients were classified as (**1**) improved, meaning that the patient’s diagnosis had improved, or (**2**) not improved, meaning that the patient’s diagnosis had not changed and was still in UWS/VS or MCS- state. The significance level was set at *p* = 0.05. In addition, we performed a probabilistic statistical analysis on the accuracy of P300 detection in patients with ’improved’ and ’not improved’, TBI and NTBI using a Gaussian distribution to validate our conclusions.

### 2.9. Data Availability

The data supporting this study are available on reasonable request but cannot be made public due to ethical protocol requirements and the sensitivity of patient data.

## 3. Results

### 3.1. CNN Results in 18 DOC Patients

By analyzing the EEG signals (including p300 signals and non-P300 signals) of 18 patients with disorders of consciousness, we found that patients with higher responsiveness to P300 (i.e., higher accuracy of the P300 detection) also had better recovery effects in later clinical treatment (this is mainly reflected in their CRS-R score).

**Figure 2:**
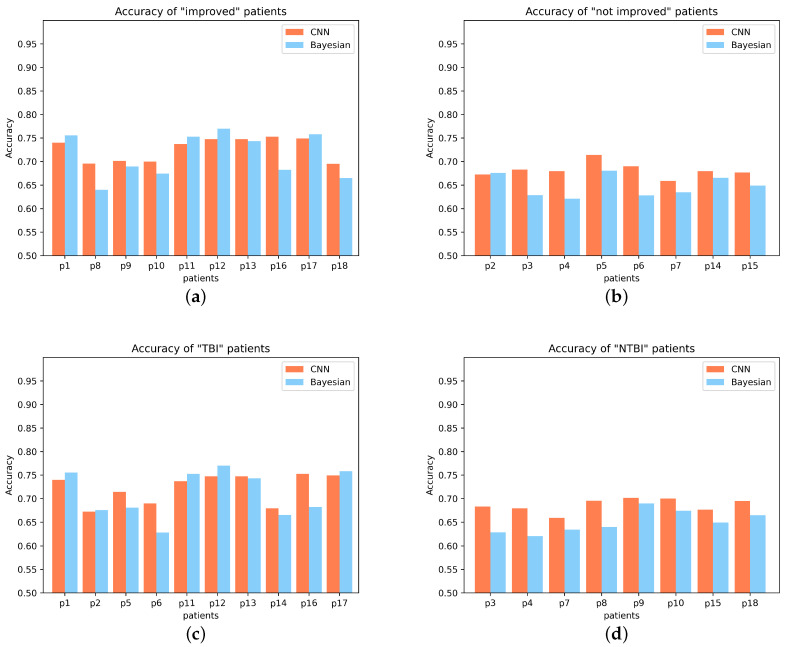
Upper panel: (**a**,**b**) represent the P300 detection accuracy of “improved” patients and “not improved” patients, respectively; (**c**,**d**) represent the P300 detection accuracy of TBI patients and NTBI patients, respectively.

**Figure 3:**
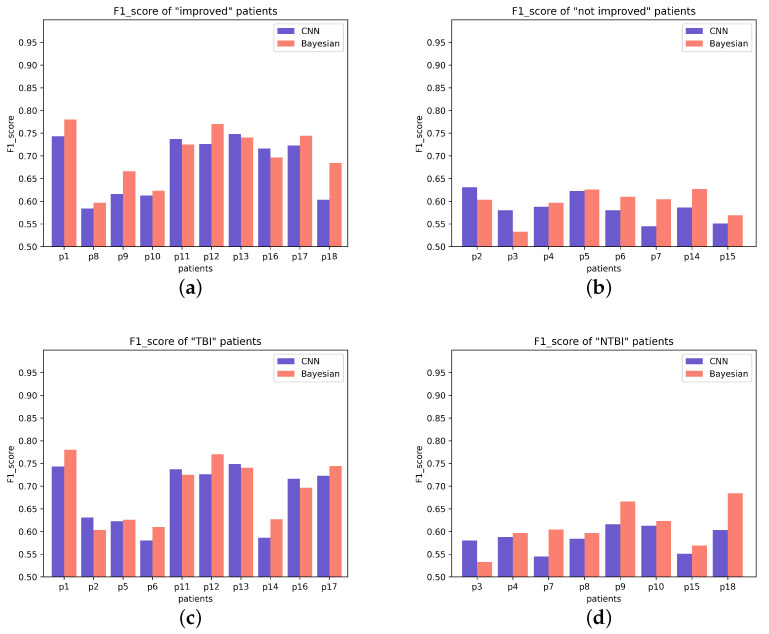
Upper panel: (**a**,**b**) represent the F1_score of “improved” patients and “not improved” patients, respectively; (**c**,**d**) represent the F1_score of TBI patients and NTBI patients, respectively.

**Figure 4:**
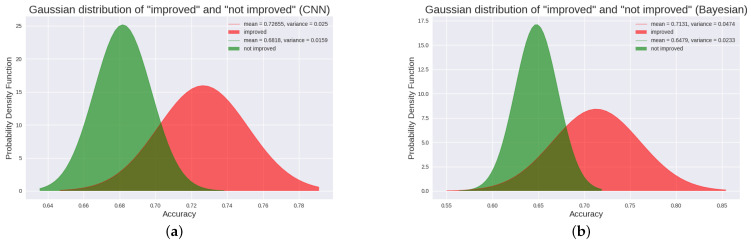
Panel (**a**) depicts the Gaussian distribution between “improved” patients and “not improved” patients using the CNN method. Panel (**b**) depicts the Gaussian distribution between “improved” patients and “not improved” patients using the Bayesian method. The probability distribution of “improved” patients and “not improved” patients. The red one is the Gaussian distribution fitted to the P300 detection accuracy of the “improved” patients, and the green one is the Gaussian distribution fitted to the P300 detection accuracy of the “not improved” patients. From the figure, we can see that there is a significant difference in the P300 detection accuracy of the two types of patients, and the P300 detection accuracy of the “improved” patients is significantly higher than that of the “not improved” patients.

For these 18 patients, patients with and without an upgrade in their level of consciousness were classified as “improved” (from UWS to MCS-, from UWS to MCS+ or from MCS- to MCS+) and “not improved” (stay in UWS or MCS-), respectively. Statistically, patients with “improved” had better outcomes than patients with “not improved” (Student‘s *t*-test, *p* = 0.00046, FDR corrected). The difference in P300 detection accuracy between “improved” and “not improved” is plotted in Figure 2a,b. From the Gaussian probability distribution plot, we can clearly see the difference between “improved” patients and “not improved” patients. In addition, we performed a statistical analysis of the F1_score of 18 patients. We found that the F1_score of “improved” patients was significantly higher than that of “not improved” patients (Student’s *t*-test, *p* = 0.0019, FDR corrected). The difference in F1_score between the two is plotted in Figure 3a,b. The Gaussian distribution (CNN) of “improved” patients and “not improved” patients is shown in Figure 4a.

**Figure 5:**
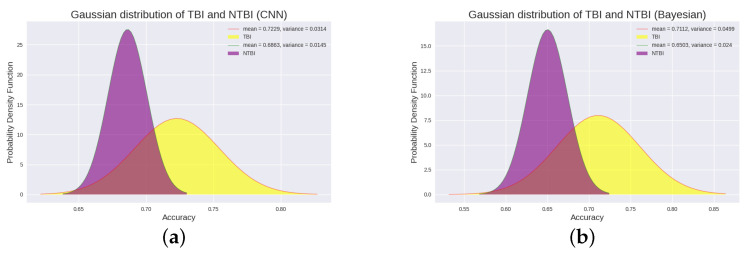
Panel (**a**) describes the Gaussian distribution between TBI patients and NTBI patients using the CNN method. Panel (**b**) depicts the Gaussian distribution between TBI patients and NTBI patients using the Bayesian method. The probability distribution of “TBI” patients and “NTBI” patients. The yellow one is the Gaussian distribution fitted to the P300 detection accuracy of the “TBI” patients, and the purple one is the Gaussian distribution fitted to the P300 detection accuracy of the “NTBI” patients. From the probability distribution plot, we found that the P300 signal differed between TBI patients and NTBI patients. This is also an important manifestation of P300-based technology for classifying TBI patients and NTBI patients.

Most interestingly, the two patients (patients P1 and P13) with high accuracy in the P300 assay significantly improved their level of consciousness after the experiment (either from UWS to MCS+ or from MCS- to MCS+). Specifically, their CRS-R scores improved from 5 and 9 (before the experiment) to 9 and 19 (after the experiment), respectively. This suggests that patients with higher levels of consciousness are more likely to have P300 detected. In addition, we specifically analyzed P300 detection accuracy in TBI patients and NTBI patients (See Figure 2c,d for details). We found that TBI patients had significantly higher P300 detection accuracy than NTBI patients (Student’s *t*-test, *p* = 0.0078, FDR corrected). The Gaussian distribution (CNN) of “TBI” patients and “NTBI” patients is shown in Figure 5a. This picture clearly shows the difference between TBI patients and NTBI patients. Moreover, in order to better evaluate our experimental results, we introduced the F1_score to evaluate the results. We performed statistical analysis on the F1_score of TBI and NTBI patients and found that the F1_score of TBI patients was significantly higher than that of NTBI patients (Student’s *t*-test, *p* = 0.0017, FDR corrected). The difference in F1_score between the two is plotted in Figure 3c,d. The P300 detection accuracy (CNN) and F1_score for each patient (including 10 UWS and 8 MCS-) are shown in Table 3. In addition, additional evaluation metrics (CNN) are shown in Table 4. It can be seen from the evaluation indicators in Table 4 that our CNN model has a good classification effect, and the obtained P300 detection accuracy has good feasibility for the classification of DOC patients.

**Figure 6:**
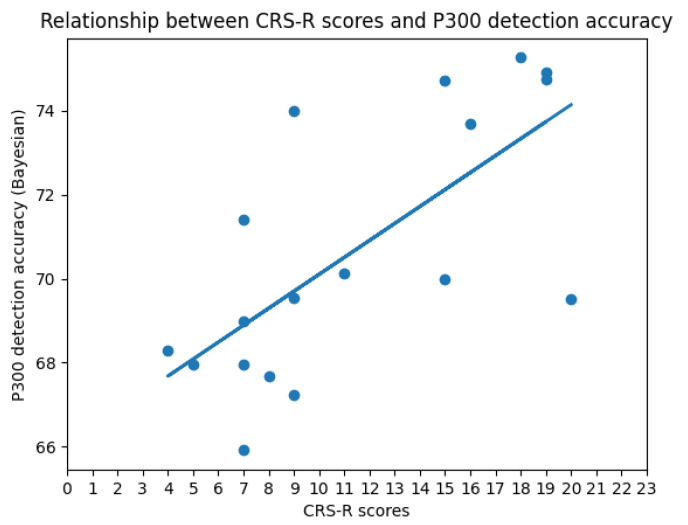
The graph shows the relationship (the linear correlation coefficient is 0.695253) between the CRS-R score and the accuracy of P300 detection (**CNN**) at a three-month follow-up. The horizontal axis is the patient’s CRS-R score, and the vertical axis is the corresponding P300 detection accuracy (**CNN**).

During our research, we were surprised to find a positive linear relationship (the linear correlation coefficient is 0.695253) between the CRS-R score and P300 detection accuracy at a three-month follow-up. That is to say, the higher the detection accuracy of P300, the higher the subsequent CRS-R score. This also confirms the conclusion of our study: there is a significant difference in the P300 detection accuracy between “improved” patients and “not improved” patients, and P300 can be an important predictor of the prognosis of DOC patients. The relationship between the CRS-R scores and P300 detection accuracy (CNN) is shown in Figure 6.

### 3.2. Bayesian Results in 18 DOC Patients

To further optimize our experiments, we introduce a Bayesian approach. The Bayesian method can also obtain the above conclusions. In the Bayesian method, the P300 detection accuracy of “improved” patients is also significantly higher than that of “not improved” patients (Student’s *t*-test, *p* = 0.0027, FDR corrected), which indicates that the difference in P300 detection accuracy of DOC patients in CNN is not accidental. The difference comparison of P300 detection accuracy is shown in Figure 2a,b. Additionally, for F1_score, “improved” patients had significantly higher F1_score than “no improvement” patients (Student’s *t*-test, *p* = 0.0004, FDR corrected). The difference comparison of F1_score is shown in Figure 3a,b. The Gaussian distribution (Bayesian) of “improved” patients and ”not improved“ patients is shown in Figure 4b.

In the classification of TBI and NTBI, there was also a significant difference in P300 detection accuracy between TBI patients and NTBI patients (Student’s *t*-test, *p* = 0.0061, FDR correction). The difference comparison of P300 detection accuracy is shown in Figure 2c,d. In addition, the F1_score of TBI patients was significantly higher than that of NTBI patients (Student’s *t*-test, *p* = 0.0114, FDR corrected). The difference comparison of F1_score is shown in Figure 3c,d. In this classification, Bayesian achieves less significant results than CNN. The Gaussian distribution (Bayesian) of “TBI” patients and “NTBI” patients is shown in Figure 5b. The P300 detection accuracy (Bayesian) and F1_scores for each patient (including 10 UWS and 8 MCS-) are shown in Table 5. In addition, additional evaluation metrics (Bayesian) are shown in Table 6. It can be seen from the evaluation indicators in Table 6 that our Bayesian method has a good classification effect, and the P300 detection accuracy obtained by this method has a strong robustness for classifying DOC patients.

**Figure 7:**
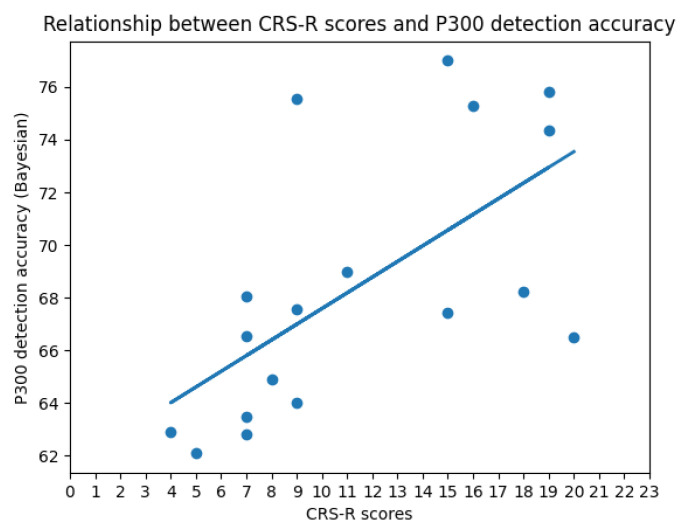
The graph shows the relationship (the linear correlation coefficient is 0.63165) between the CRS-R score and the accuracy of P300 detection (**Bayesian**) at a three-month follow-up. The horizontal axis is the patient’s CRS-R score, and the vertical axis is the corresponding P300 detection accuracy (**Bayesian**).

The positive linear relationship (the linear correlation coefficient is 0.63165) between the CRS-R scores and P300 detection accuracy (Bayesian) is shown in Figure 7. The same conclusion as above can be obtained here.

## 4. Discussion

The detection of consciousness and cognitive function in patients with DOC is highly challenging but critical to clinical care and the selection of optimal treatment options. In this study, we used DOC patients’ responsiveness to P300 to detect their consciousness. An EEG-based BCI paradigm (Audiovisual) was applied in the current study to detect patients with disorders of consciousness and then investigate their clinical outcomes. We found a significant difference (Student’s *t*-test, *p* < 0.05, FDR corrected) between the accuracy of P300 detection and subsequent recovery of consciousness in “improved” patients and “not improved” patients. There is also a significant difference in F1_score. This further supports our view. This suggests that patients with a greater likelihood of subsequent recovery have relatively higher P300 detection accuracy, revealing the existence of P300 as a specific predictor of conscious behavioral recovery. The findings showed that, in the audiovisual paradigm, P300 was highly correlated with subsequent recovery in DOC patients. Through the P300 factor, our study provides valid evidence for knowledge of prognostic parameters and supports the use of P300 in clinical practice to predict the likelihood of recovery in patients with DOC. Moreover, our study also found a linear relationship between the CRS-R score and the P300 detection accuracy: with the improvement of the CRS-R score, the P300 detection accuracy also increased, which once again confirmed that the P300 was useful in predicting patients with DOC of prognostic value. In addition, we also found a significant difference in the accuracy of P300 detection between TBI patients and NTBI patients (Student’s *t*-test, *p* < 0.05, FDR corrected). It is generally accepted that patients who received traumatic brain injury had inferior ERP responses to those who did not, but our results suggest that P300 detection in patients with TBI is not necessarily less accurate than in patients with NTBI (P2). Conversely, the prognosis of TBI patients is not necessarily poor. In our experiment, 6 out of 10 TBI patients had an improvement in their diagnosis after three months and were classified as “improved”. The results of this study provide an important direction for further research. DOC exists on a temporal continuum, and the principles and confounders of assessment, prognosis, and treatment have changed over time [31]. A recent study classified disturbances of consciousness into acute, subacute, and chronic periods [32]. The acute phase includes time spent at the site of the injury, in the emergency room and intensive care unit (ICU), while the subacute and chronic phases extend to time spent in hospitalization and recovery. This study defined the acute phase of DOC as the first 28 days after injury, followed by the subacute-chronic phase. It is generally believed that patients with shorter injury durations tend to be more likely to regain consciousness. However, in our study, patients with a longer duration of injury (in the subacute-chronic phase) did not necessarily have a worse prognosis (P1, P8). Conversely, patients with a shorter duration of injury (in the acute phase) did not necessarily have a better prognosis (P5). Due to the small sample size of our current study, a relationship between injury time and prognosis cannot be drawn. We will select more representative DOC samples for further research.

In our study, the most important is the P300 characteristic. At present, P300 signal features have been widely used in clinical medicine to diagnose patients. In [33], the authors used a combination of exploratory eye movements (EEMs) and P300 to build a model to detect depression and predict efficacy. In [34], to elucidate the functional significance of P300 in depression, the authors examined whether initial P300 amplitude prospectively predicted changes in depressive symptoms in a community sample of 58 adults with current depression. Their results showed that, even after controlling for initial depression, a reduction in P300 magnitude at initial visit was associated with higher total depressive symptoms at follow-up. In [35], Liu et al. explored the role of nonlinear dynamic analysis of EEG in predicting the prognosis of patients with UWS and MCS. In [36], Pan et al. proposed a hybrid BCI system combining P300 potential and emotional patterns to improve the performance of consciousness detection. They asked patients to perform corresponding tasks to demonstrate their remaining awareness and emotion-related abilities. All these indicate that the P300-based BCI system has played an important role in the clinical prediction of patient types.

In recent years, an increasing number of researchers have focused on finding key factors for the prognosis of patients with DOC, and many new techniques have been explored, including EEG [37] and resting-state functional magnetic resonance imaging (rs-fMRI) [38], which have had a huge impact on research on disorders of consciousness. In addition, functional near-infrared spectroscopy (fNIRS) has recently attracted a lot of attention for non-invasive BCI. In [39], the authors’ study aimed to address the inherent delay in the hemodynamic responses (HRs) for the command generation time. For this, HRs in the sensorimotor cortex were evaluated for the fNIRS-based BCI. There are also many studies of EEG application cases. In [40], Hussain et al. investigated three-channel EEG sleep recordings of 154 people and found that the delta wave power ratio can be considered as a biomarker for different sleep stages. It indicates that the EEG-based sleep stage prediction method is expected to be used in wearable sleep monitoring systems. In addition, by investigating the neural responses of 17 healthy male drivers, they also found that delta–alpha ratio (DAR) and delta–theta ratio (DTR) showed a strong correlation with a resting state, city-roadways driving state, and expressway driving state [41]. These findings indicate that EEG-based prediction methods have been widely used in various life scenarios. However, in practice, due to the safety issues of implanted devices, there are certain safety risks when performing fMRI. It also highlights the advantages of ERP as a more widely used and practical technology. ERPs are special evoked potentials that are recorded on the surface of the skull when a person is cognitively processing a specific stimulus. It mainly includes two kinds of exogenous components and endogenous components. Endogenous ERPs are significantly different from exogenous stimulus-related potentials. The exogenous component is affected by the physical properties of the stimulus, while the endogenous component is not affected by the physical properties of the stimulus, and is related to the subject’s level of consciousness and attention. P300 is the third positive wave of ERP, which is an endogenous component that is not affected by physical properties (shape, size, vision, hearing, etc.) stimulation, and is closely related to cognitive function [42]. It is very suitable for us to test the level of consciousness of patients with DOC.

Because most patients with DOC have lost their motor ability and cannot speak, they can only communicate with the outside world through eye movements and blinking. It is difficult to collect EEG data with a complex paradigm like a normal person. Therefore, the EEG data collection paradigm for patients with DOC should be as concise as possible, and it is even best if it can be induced without an interface. In clinical practice, we should choose the stimulation paradigm according to the cognitive function of different patients. Using a multimodal approach is a good solution. In [43], Li et al. proposed to share the control system as much as possible to reduce user workload. In a multimodal BCI system, multiple brain modalities, sensory modalities, signal inputs, or intelligent technologies can be combined. They propose to consider the possibility of personalized design, especially when designing multimodal BCI systems for a specific group of patients. In particular, the spatial, temporal, and semantic coherence of audiovisual stimuli needs to be ensured to evoke stronger brain patterns. The audio-visual combination was stronger than the brain patterns evoked by auditory-only or visual-only stimuli. The experimental paradigm of audio-visual integration we adopted is very convenient and does not require subjects to do many physical movements. P300 responses were stimulated by simultaneous visual and auditory stimulation by simply watching the flashes on the screen and listening to the corresponding numbers read out from the speakers. For patients with DOC who have lost motor function, the entire experimental process can be completed relatively easily without adding too much burden.

A comparison of our study and other studies is presented in Table 7. The study by Zhang et al. [44] mainly demonstrated a statistical relationship between the presence of P300 and prognosis. Their study only judged the existence of consciousness level of DOC patients by the presence or absence of P300, and there was no specific index to judge the level of consciousness. Rosseal et al. [45] found that delta-band modulation is related to the generation of the P300. This revealed key pathological changes in DoC and provided a new fine-grained marker of residual cognitive function. In addition, our method is to distinguish the two types of patients by finding the difference in the P300 detection accuracy of the two. In addition, we also found a positive correlation between the CRS-R score and the P300 detection accuracy. Although the correlation is not high at present, it provides a general direction for our follow-up research. For clinical diagnosis, the acquisition of P300 signal is more convenient, especially for DOC patients, who lack exercise ability and cannot perform too many difficult experiments. If we can predict the prognosis of patients through P300 detection accuracy, this will be a major breakthrough for the clinical diagnosis of DOC. It is clear that our research approach is more feasible for DOC patients and has a greater potential for breakthroughs.

At present, the clinical research on P300 is the most extensive. If we simply use CRS-R score to detect the patients’ level of consciousness, it is easy to cause misdiagnosis and delay the optimal treatment period for the patients. Early detection of awareness is associated with prognosis [46,47,48], and is a major determinant of clinical nursing decision goals [49]. The disadvantage of CRS-R is that it is time-intensive, and the entire evaluation time may take up to 30–40 min [50]. Therefore, if the P300 is used as an auxiliary diagnosis, it can greatly improve the detection efficiency of the patient’s level of consciousness, help the nursing staff to formulate a more appropriate treatment plan to a large extent, and improve the possibility of the patient’s recovery of consciousness. In the study of Zhang et al., they demonstrated that P300 is potentially a surrogate tool or predictor for predicting the likelihood of recovery in DOC patients [44]. Cavinato et al. found a correlation between stimulus complexity and increased P300 latency in MCS patients and healthy controls, but not in UWS patients [51]. Annen and colleagues studied P300 responses to auditory and vibrotactile stimuli. When comparing UWS with MCS patients, and even when comparing TBI patients with NTBI patients, they found no differences in the P300 signature [52]. Investigating P300 responses in relation to the CRS-R score showed that the magnitude of the novelty P300 correlates with the CRS-R and even with the auditory subscore [53]. These findings also coincide with our findings. Surprisingly, in our study, we found not only differences in P300 detection accuracy between TBI patients and NTBI patients, but also P300 differences between “improved” and “not improved” patients.

## 5. Conclusions

In conclusion, the current clinical significance of our findings is that P300 can be used to effectively classify patients with better or worse recovery of consciousness at a later stage, and predict patients who are more likely to recover, revealing the potential prognostic value of P300. Currently, there are many people with disorders of consciousness worldwide. They may be chronically vegetative, or MCS, for a long time. Without an accurate identification method to determine which patients are more likely to regain consciousness, medical power is not sufficient to support the treatment of so many patients, and many patients who have the opportunity to regain consciousness will miss the best time. The findings presented in this study can make doctors and caregivers predict the likelihood of improvement in these patients and optimize their treatment. In addition, we believe that combining clinical approaches with neuroimaging, along with factors such as etiology and time of injury, will help to identify more reliable predictors and provide new insights into the prognosis of patients with DOC.

## Figures and Tables

**Table 1:** Information and CRS-R scores for all patients.

							CRS-R Scores (Subscores)	
Patients	Age (Years Old)	Gender	Etiology	Time Since Injury (Months)	Diagnosis (Before)	Diagnosis (After)	Before the Experiment	After the Experiment
P1	43	M	TBI	5	UWS	MCS+	5 (1-0-1-1-0-2)	9 (3-1-2-1-0-2)
P2	51	M	TBI	20	UWS	UWS	9 (2-1-2-2-0-2)	9 (2-1-2-2-0-2)
P3	29	M	ABI	8.5	UWS	UWS	4 (1-0-1-0-0-2)	4 (1-0-1-0-0-2)
P4	37	M	ABI	2	UWS	UWS	5 (0-0-2-1-0-2)	5 (0-0-2-1-0-2)
P5	38	M	TBI	1	UWS	UWS	7 (1-1-2-1-0-2)	7 (1-1-2-1-0-2)
P6	33	M	TBI	2	UWS	UWS	7 (1-0-2-2-0-2)	7 (1-0-2-2-0-2)
P7	40	M	ABI	2	UWS	UWS	5 (1-0-2-0-0-2)	7 (1-0-2-2-0-2)
P8	52	M	CVD	4.5	UWS	MCS-	5 (1-1-0-1-0-2)	9 (2-3-0-2-0-2)
P9	42	M	CVD	4	UWS	MCS+	7 (1-1-2-1-0-2)	11 (3-2-3-1-0-2)
P10	26	M	CVD	1	UWS	MCS+	7 (1-1-2-1-0-2)	15 (4-5-2-1-1-2)
P11	48	M	TBI	3.5	MCS-	MCS+	12 (1-2-5-1-0-2)	16 (3-3-5-2-1-2)
P12	34	M	TBI	1.5	MCS-	MCS+	9 (1-1-5-1-0-1)	15 (4-4-5-1-0-1)
P13	37	M	TBI	4	MCS-	MCS+	9 (1-3-2-1-0-2)	19 (3-5-6-2-1-2)
P14	20	M	TBI	4	MCS-	MCS-	7 (1-0-3-1-0-2)	7 (1-0-3-1-0-2)
P15	19	M	ABI	1.5	MCS-	MCS-	8 (1-1-3-1-0-2)	8 (1-1-3-1-0-2)
P16	17	M	TBI	2	MCS-	MCS+	8 (1-1-3-1-0-2)	18 (4-5-3-1-2-3)
P17	46	F	TBI	1.5	MCS-	MCS+	7 (1-0-3-1-0-2)	19 (3-5-6-2-1-2)
P18	46	M	CVD	2	MCS-	MCS+	9 (1-1-4-1-0-2)	20 (4-5-6-2-1-2)

**Note**: Coma Recovery Scale-Revised(CRS-R) subscales: auditory, visual, motor, oromotor, communication, and arousal functions. F: Female; M: Male ABI: acquired brain injury; CVD: cerebrovascular disease.

**Table 2:** EEG sample information for all patients.

Patients	Total Number of Samples	Number of P300	Number of Non-P300
P1	1100	550	550
P2	900	450	450
P3	2880	1395	1485
P4	2880	1398	1482
P5	2880	1376	1504
P6	2880	1387	1493
P7	2880	1378	1502
P8	3400	1647	1753
P9	2880	1388	1492
P10	2880	1389	1491
P11	1080	540	540
P12	1100	550	550
P13	760	380	380
P14	1100	550	550
P15	2880	1379	1501
P16	3000	1454	1546
P17	2880	1386	1494
P18	2880	1389	1491

**Table 3:** Accuracy and F1_score of the P300 detection (**CNN**) for all patients.

Patients	Age (Years Old)	Etiology	Time Since Injury (Months)	Diagnosis (Before)	Diagnosis (After)	Improved/Not Improved	Accuracy Rate	F1_SCORE
P1	43	TBI	5	UWS	MCS+	improved	**74.00%**	0.7436
P2	51	TBI	20	UWS	UWS	not improved	67.22%	0.6308
P3	29	ABI	8.5	UWS	UWS	not improved	68.30%	0.5803
P4	37	ABI	2	UWS	UWS	not improved	67.95%	0.5882
P5	38	TBI	1	UWS	UWS	not improved	71.42%	0.6224
P6	33	TBI	2	UWS	UWS	not improved	68.99%	0.5800
P7	40	ABI	2	UWS	UWS	not improved	65.91%	0.5452
P8	52	CVD	4.5	UWS	MCS-	improved	**69.55%**	0.5843
P9	42	CVD	4	UWS	MCS+	improved	**70.14%**	0.6160
P10	26	CVD	1	UWS	MCS+	improved	**70.00%**	0.6130
P11	48	TBI	3.5	MCS-	MCS+	improved	**73.70%**	0.7373
P12	34	TBI	1.5	MCS-	MCS+	improved	**74.73%**	0.7262
P13	37	TBI	4	MCS-	MCS+	improved	**74.74%**	0.7485
P14	20	TBI	4	MCS-	MCS-	not improved	67.95%	0.5863
P15	19	ABI	1.5	MCS-	MCS-	not improved	67.67%	0.5512
P16	17	TBI	2	MCS-	MCS+	improved	**75.27%**	0.7162
P17	46	TBI	1.5	MCS-	MCS+	improved	**74.91%**	0.7230
P18	46	CVD	2	MCS-	MCS+	improved	**69.51%**	0.6033

**Note**: The P300 detection accuracy of “improved” patients is in bold. ABI: acquired brain injury; CVD: cerebrovascular disease.

**Table 4:** Additional evaluation metrics (**CNN**) for all patients.

Patients	Precision	Sensitivity	Specificity	NPV	AUPRC	F${}_{0.5}$	F${}_{2}$
P1	0.7854	0.7836	0.6964	0.8141	0.8386	0.7564	0.7590
P2	0.7336	0.6022	0.7422	0.6918	0.7673	0.6806	0.6066
P3	0.8041	0.4558	0.8964	0.6376	0.7617	0.6958	0.4984
P4	0.7833	0.4719	0.8754	0.6374	0.7558	0.6911	0.5124
P5	0.8462	0.4926	0.9170	0.6638	0.7906	0.7396	0.5374
P6	0.8198	0.4511	0.9117	0.6429	0.7676	0.7023	0.4949
P7	0.7654	0.4242	0.8798	0.6199	0.7342	0.6586	0.4655
P8	0.8067	0.4613	0.9102	0.6511	0.7628	0.6984	0.5034
P9	0.8392	0.4935	0.8950	0.6525	0.7884	0.7308	0.5353
P10	0.8258	0.4888	0.8969	0.6518	0.7805	0.7246	0.5317
P11	0.7892	0.7044	0.7037	0.8084	0.8372	0.7551	0.7485
P12	0.8172	0.7200	0.7745	0.7995	0.8386	0.7675	0.7141
P13	0.7958	0.7868	0.7079	0.8275	0.8446	0.7637	0.7626
P14	0.7918	0.4697	0.8743	0.6379	0.7584	0.6930	0.5098
P15	0.7909	0.4334	0.9023	0.6362	0.7485	0.6665	0.4734
P16	0.8465	0.6727	0.8327	0.7693	0.8414	0.7801	0.6822
P17	0.8108	0.7164	0.7818	0.8006	0.8345	0.7632	0.7108
P18	0.8176	0.4801	0.8955	0.6486	0.7742	0.7151	0.5226

**Note**: AUPRC: The area under the precision–recall curve; NPV: negative predictive value.

**Table 5:** Accuracy of the P300 detection (**Bayesian**) for all patients.

Patients	Age (Years Old)	Etiology	Time Since Injury (Months)	Diagnosis (Before)	Diagnosis (After)	Improved/Not Improved	Accuracy Rate	F1_SCORE
P1	43	TBI	5	UWS	MCS+	improved	**75.55%**	0.7803
P2	51	TBI	20	UWS	UWS	not improved	67.56%	0.6035
P3	29	ABI	8.5	UWS	UWS	not improved	62.88%	0.5327
P4	37	ABI	2	UWS	UWS	not improved	62.08%	0.5966
P5	38	TBI	1	UWS	UWS	not improved	68.06%	0.6259
P6	33	TBI	2	UWS	UWS	not improved	62.81%	0.6099
P7	40	ABI	2	UWS	UWS	not improved	63.47%	0.6046
P8	52	CVD	4.5	UWS	MCS-	improved	**64.00%**	0.5968
P9	42	CVD	4	UWS	MCS+	improved	**68.96%**	0.6663
P10	26	CVD	1	UWS	MCS+	improved	**67.43%**	0.6234
P11	48	TBI	3.5	MCS-	MCS+	improved	**75.28%**	0.7252
P12	34	TBI	1.5	MCS-	MCS+	improved	**77.00%**	0.7702
P13	37	TBI	4	MCS-	MCS+	improved	**74.34%**	0.7403
P14	20	TBI	4	MCS-	MCS-	not improved	66.53%	0.6269
P15	19	ABI	1.5	MCS-	MCS-	not improved	64.90%	0.5692
P16	17	TBI	2	MCS-	MCS+	improved	**68.23%**	0.6966
P17	46	TBI	1.5	MCS-	MCS+	improved	**75.82%**	0.7443
P18	46	CVD	2	MCS-	MCS+	improved	**66.49%**	0.6842

**Note**: The P300 detection accuracy of “improved” patients is in bold. ABI: acquired brain injury; CVD: cerebrovascular disease.

**Table 6:** Additional evaluation metrics (**Bayesian**) for all patients.

Patients	Precision	Sensitivity	Specificity	NPV	AUPRC	F${}_{0.5}$	F${}_{2}$
P1	0.7310	0.9000	0.6109	0.9077	0.8405	0.7425	0.8426
P2	0.7517	0.5444	0.8067	0.6809	0.7620	0.6768	0.5606
P3	0.6846	0.4735	0.7741	0.6223	0.7065	0.6047	0.4908
P4	0.6144	0.6372	0.6053	0.6884	0.7138	0.5997	0.6138
P5	0.7520	0.5803	0.7731	0.6763	0.7665	0.6870	0.5919
P6	0.6494	0.6385	0.6195	0.6687	0.7311	0.6240	0.6193
P7	0.6450	0.6211	0.6473	0.6856	0.7237	0.6211	0.6078
P8	0.6668	0.5864	0.6904	0.6496	0.7267	0.6262	0.5863
P9	0.6969	0.6905	0.6882	0.7472	0.7682	0.6768	0.6748
P10	0.7192	0.6017	0.7418	0.7037	0.7564	0.6689	0.6033
P11	0.8221	0.7093	0.7963	0.7876	0.8384	0.7696	0.7084
P12	0.8536	0.7600	0.7800	0.7915	0.8668	0.8098	0.7561
P13	0.7388	0.8158	0.6711	0.8576	0.8234	0.7281	0.7777
P14	0.7114	0.6118	0.7150	0.6938	0.7550	0.6668	0.6107
P15	0.7171	0.5146	0.7758	0.6512	0.7335	0.6381	0.5298
P16	0.6773	0.7861	0.5872	0.7745	0.7829	0.6763	0.7412
P17	0.7989	0.7582	0.7582	0.8147	0.8390	0.7669	0.7455
P18	0.6405	0.7796	0.5581	0.7620	0.7632	0.6521	0.7343

**Note**: AUPRC: The area under the precision–recall curve; NPV: negative predictive value.

**Table 7:** A comparison of our study and other studies.

Study	Time Since Injury	Etiology	Diagnosis	Results	Conclusions
Zhang et al. [44]	1.6–21 months	8 TBI, 10 NTBI	2 coma, 9 VS/UWS, 7 MCS	All of MCS and four out of nine UWS/VS showed an intact P300 in 8 paradigm. All of the five patients with P300 in both paradigms were finally awake after 12 months, while none of the eight patients without P300 regained consciousness.	A highly significant relationship between P300 and subsequent recovery was found.
Rosseal et al. [45]	35–360 days	3 TBI, 10 NTBI	13 UWS	4 UWS patients demonstrated clear EEG-based indices of task following in one or both paradigms, which did not correlate with clinical factors. The efficacy of somatosensory discrimination strongly correlated with the clinical outcome at 6 months. The BCI system also yielded the expected results with healthy controls.	Neurophysiological correlates of somatosensory discrimination can be detected in clinically unresponsive patients and are associated with recovery of behavioral responsiveness at six months.
**Ours**	1–20 months	10 TBI, 8 NTBI	10 VS/UWS, 8 MCS	The results showed that the P300 detection accuracy of “improved” patients and “not improved” patients, TBI patients and NTBI patients was significantly different (*p* < 0.05). Moreover, there was a positive correlation between CRS-R score and P300 detection accuracy.	The current clinical significance of our findings is that P300 can be used to effectively classify patients with better or worse recovery of consciousness at a later stage, and predict patients who are more likely to recover, revealing the potential prognostic value of P300.

## Data Availability

The original contributions presented in the study are included in the article material, and further inquiries can be directed to the corresponding author/s.

## Abbreviations

The following abbreviations are used in this manuscript:

DOC	Disorders Of Consciousness
UWS	Unresponsive Wakefulness Syndrome
VS	Vegetative State
MCS	Minimally Conscious State
MCS+	Minimally Conscious State Plus
MCS-	Minimally Conscious State Minus
ERP	Event Related Potential
ERPs	Event Related Potentials
TBI	Traumatic Brain Injury
NTBI	Non-Traumatic Brain Injury
LIS	Locked-In Syndrome
CRS-R	Coma Recovery Revised-Scale
BCI	Brain–Computer Interface
BCIs	Brain–Computer Interfaces
EEG	Electroencephalogram
CNN	Convolutional Neural Network
CNNs	Convolutional Neural Networks
SSVEPs	Steady state visually evoked potentials
SVM	Support Vector Machines
LDA	Linear Discriminant Analysis
